# Miniaturized silicon-based capacitive six-axis force/torque sensor with large range, high sensitivity, and low crosstalk

**DOI:** 10.1038/s41378-024-00831-0

**Published:** 2024-11-29

**Authors:** Renjie Tan, Yong Xia, Xiangguang Han, Linya Huang, Wendi Gao, Chen Jia, Ping Yang, Qijing Lin, Shujiang Ding, Chenying Wang, Libo Zhao

**Affiliations:** 1 State Key Laboratory for Manufacturing Systems Engineering, International Joint Laboratory for Micro/Nano Manufacturing and Measurement Technologies, Xi’an Jiaotong University (Yantai) Research Institute for Intelligent Sensing Technology and System, Xi’an Jiaotong University, 710049 Xi’an, China; 2 School of Mechanical Engineering, Xi’an Jiaotong University, Xi’an, 710049 China; 3 School of Instrument Science and Technology, Xi’an Jiaotong University, Xi’an, 710049 China; 4 Electronic Materials Research Laboratory, Key Laboratory of the Ministry of Education, School of Electronic Science and Engineering, Xi’an Jiaotong University, 710049 Xi’an, China; 5 Shandong Laboratory of Yantai Advanced Materials and Green Manufacturing, Yantai, 265503 China; 6 Engineering Research Center of Energy Storage Materials and Devices of Ministry of Education, National Innovation Platform (Center) for Industry-Education Integration of Energy Storage Technology, School of Chemistry, Xi’an Jiaotong University, Xi’an, 710049 China

**Keywords:** Electrical and electronic engineering, Sensors

## Abstract

Miniaturized six-axis force/torque sensors have potential applications in robotic tactile sensing, minimally invasive surgery, and other narrow operating spaces, where currently available commercial sensors cannot meet the requirements because of their large size. In this study, a silicon-based capacitive six-axis force/torque sensing chip with a small size of 9.3 × 9.3 × 0.98 mm was designed, fabricated, and tested. A sandwich decoupling structure with a symmetrical layered arrangement of S-shaped beams, comb capacitors, and parallel capacitors was employed. A decoupling theory considering eccentricity and nonlinear effects was derived to realize low axial crosstalk. The proposed S-shaped beams achieved a large measurement range through stress optimization. The results of a coupled multiphysics field finite-element simulation agreed well with those of theoretical analyses. The test results show that the proposed sensing chip can detect six-axis force/torque separately, with all crosstalk errors less than 2.59%FS. Its force and torque measurement ranges can reach as much as 2.5 N and 12.5 N·mm, respectively. The sensing chip also has high sensitivities of 0.52 pF/N and 0.27 pF/(N·mm) for force and torque detection, respectively.

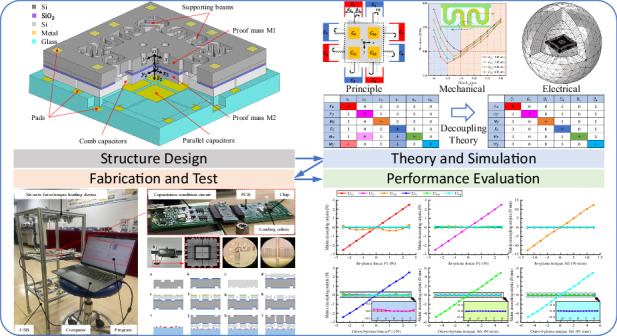

## Introduction

Force and torque in all directions can be detected simultaneously using six-axis force/torque sensors for condition monitoring, scientific research, and feedback control in such applications as aerospace, industrial automation, intelligent robotics, and biomedicine^1–4^.

The market for six-axis force/torque sensors is dominated by products from ATI, Inc. and HBM, Inc., because of their excellent overload and accuracy. Elastomers are manufactured using precision machining, and metallic-strain gauges are precisely arranged on the elastomers to form multiple Wheatstone bridges to output the decoupled information. Various six-axis force/torque sensors based on this metallic-strain principle have been developed^5,6^. However, these sensors are too large for force/torque detection in a narrow space. Moreover, the cost of these sensors is extremely high and cannot be lowered significantly because of the limitations of batch manufacturing.

Silicon-based microelectromechanical system (MEMS) six-axis force/torque sensors fabricated using silicon microfabrication technology have the advantages of miniaturization and batch manufacturing, which are ideal for solving problems of large size and high cost. Compared with flexible tactile sensors^7–10^, which can also be small, silicon-based MEMS sensors exhibit better dynamic performance, lower hysteresis, and smaller crosstalk. Piezoresistive and capacitive principles are most commonly used in the structural design of silicon-based MEMS six-axis force/torque sensors because of their easier decoupling than other principles, such as resonant^11^ and piezoelectric.

The decoupling principle of MEMS piezoresistive six-axis force/torque sensors is similar to that of commercial metallic-strain-type sensors. The cross beam^12–15^, T-shaped beam^16^, or separated straight beam^17,18^ are fabricated by a deep-etching process, the piezoresistors are arranged on the surface or sides of supporting beams by ion implantation^12,16,17^ or ion thermal diffusion^14,18^ processes, and multiple Wheatstone bridges are formed by piezoresistors to output the decoupled information. These sensors have higher sensitivity than commercial sensors because of the small elastomers and highly sensitive piezoresistors. However, the fabrication of confined and electrically stable piezoresistors is complicated, and the output is easily affected by temperature.

Silicon-based capacitive MEMS six-axis force/torque sensors work with electrostatic effects and are manufactured mainly by deep reactive ion etching (DRIE) and bonding processes. These sensors have a simpler manufacturing process, better temperature independence, and higher sensitivity than piezoresistive MEMS sensors. Beyeler et al. developed a MEMS six-axis force/torque sensor with micronewton and newton nanometer resolution^19,20^. The in-plane and out-of-plane forces/torques are detected by comb and parallel capacitors. However, complex algorithm decoupling based on calibration data is necessary to achieve low crosstalk, and it is only suitable for detecting a 1-mN weak force and 2.6-mN·mm torque in such applications as cell monitoring. Alveringh et al. developed a MEMS five-axis force/torque sensor with a newton and newton millimeter range^21^. The sensor fabrication process is simple. V-shaped beams and comb capacitors are obtained by a two-step deep-etching process on the substrate layer and device layer of an silicon-on-insulator wafer, respectively. However, the chip cannot detect the in-plane torque, and the values of the crosstalk errors were not mentioned. Brookhuis et al. developed some six-axis force/torque sensing chips with a newton and newton millimeter range^22,23^. Silicon pillars fabricated by DRIE were used as the bearing structure, and area-variable and space-variable parallel capacitors fabricated by silicon–silicon bonding were used to detect the in-plane and out-of-plane force/torque, respectively. However, the crosstalk for in-plane torque *Mz* detection was not analyzed and tested, and the crosstalk errors of less than 2.8%FS were not for all 30 coupling cases. Moreover, the sensitivity of the in-plane force detection was too low. Overall, current silicon-based capacitive MEMS six-axis force/torque chips were rarely analyzed and tested to evaluate all crosstalk errors. Most of them have serious crosstalk because of the complexity of the decoupling structure design and decoupling theory.

Herein, a sandwich-type MEMS six-axis force/torque sensing chip is proposed based on the capacitive principle. It adopts a decoupling structure with S-shaped beams, comb capacitors, and parallel capacitors arranged symmetrically in layers. The decoupling theory established considering eccentricity and nonlinear effects further suppresses the crosstalk. Theoretical and finite-element simulation models verify the feasibility and optimize the performance of the proposed sensing chip. Finally, the proposed chip was fabricated, and calibration equipment was built to test the sensing chip under six-axis force/torque loadings to obtain its basic performance.

## Materials and methods

### Structure design and working principle

**Fig. 1:**
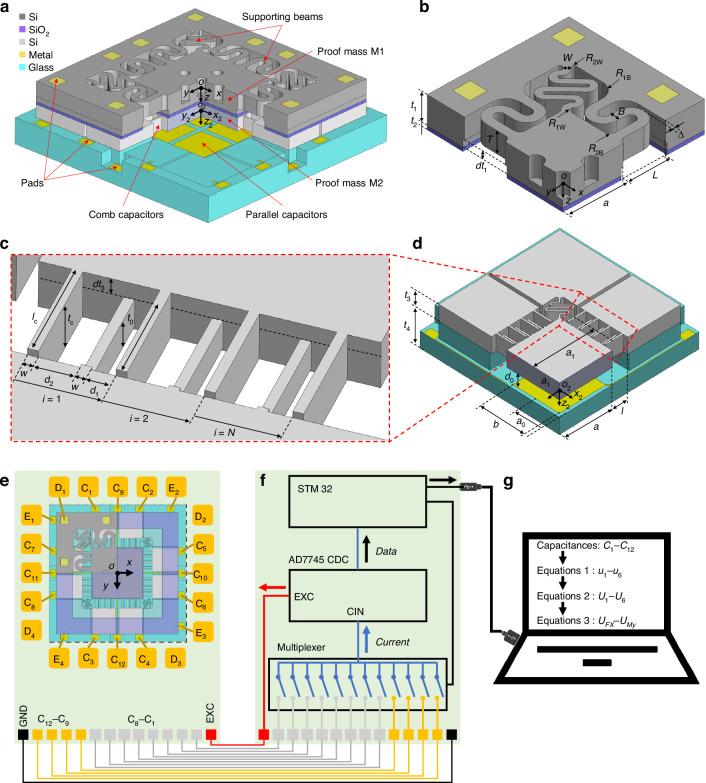
Overview of the MEMS six-axis force/torque sensing chip and its conditioning circuit. **a** Overall structure. **b** Parametric model of the S-shaped beams. **c** Parametric model of the comb capacitors. **d** Parametric model of the parallel capacitors. **e** Printed circuit board to fix chip and transfer signals. **f** Schematic of the multichannel capacitance conditioning circuit. **g** PC and program for data processing and decoupling operations

The MEMS six-axis force/torque sensing chip consists of five layers: top silicon, silicon oxide, bottom silicon, metal, and glass (Fig. 1a). The S-shaped beams and proof mass M1 in top silicon form a mass-spring system that serves as a bearing and transfers the measured loads. The comb capacitors in the bottom silicon and plate capacitors on the glass serve to sense the displacement/angle of proof mass M2. The top and bottom silicon layers are connected and electrically insulated by the silicon oxide.

First, the six-axis force/torque applied to the origin of the chip cording system *o*-*xyz* is converted to the six-axis displacement/angle of proof mass. Subsequently, the six-axis displacement/angle is converted to a change in the multichannel capacitances. The four corners and eight middle S-shaped beams are arranged symmetrically in parallel around proof mass M1 to improve the load-bearing capacity. Eight comb capacitors are symmetrically arranged around proof mass M2, and four parallel capacitors are symmetrically arranged under proof mass M2 to improve the decoupling capability for the six-axis displacement/angle. Figure 1b–d shows enlarged views of the S-shaped beams, comb capacitors, and parallel capacitors, respectively. The critical size parameters are listed in Table 1.

**Table 1:** Important parameters of six-axis force/torque sensing chip

Symbol	Parameter	Value
*a*	Half-length of proof mass	1700 μm
*a*_0_	Location of parallel capacitors	850 μm
*b*	Width of parallel capacitors	1500 μm
*d*_0_	Space of parallel capacitors	6 μm
*a*_1_	Location of combs	2000 μm
*l*_c_	Length of combs	560 μm
*l*_0_	Overlap length of combs	520 μm
*t*_0_	Overlap height of combs	68 μm
*w*	Width of combs	20 μm
*d*_1_	Space of combs	5 μm
*d*_2_	Antispace of combs	15 μm
*N*	Array number of combs	24
*W*	Width of corner S-shaped beams	175 μm
*R*_1W_	Fillet 1 of corner S-shaped beams	150 μm
*R*_2W_	Fillet 2 of corner S-shaped beams	50 μm
*B*	Width of middle S-shaped beams	250 μm
*R*_1B_	Fillet 1 of middle S-shaped beams	400 μm
*R*_2B_	Fillet 2 of middle S-shaped beams	150 μm
*T*	Thick of S-shaped beams	390 μm

Figure 1e, f shows schematics of the electrical connection between the sensing chip and the condition circuit. The excitation signal *EXC* provided by the circuit is transmitted to proof mass M2 through pads E_1_–E_4_ and the gold wire. Proof mass M2 is the common electrode of the 12 capacitors. The output signals generated by the noncommon electrodes of the 12 capacitors are transmitted to the circuit through the gold wire and pads C_1_–C_12_. The ground signal *GND* provided by the circuit is transmitted to the top silicon through pads D_1_–D_12_ and gold wires to shield the parasitic capacitance.

The 12 output signals *C*_1_–*C*_12_ are converted to digital signals by time-division multiplexing through a microcontroller (STM32, STMicroelectronics, Inc.), multiplexers, and AD7745 from Analog Devices, Inc. (Fig. 1e, f). The digital signals are transmitted to a PC for data processing and decoupling operations to obtain the individual six-axis force/torque information (Fig. 1g).

### Decoupling principle

The decoupling structure design and theoretical formulas to suppress crosstalk among the six-axis force/torque detections are discussed.

**Fig. 2:**
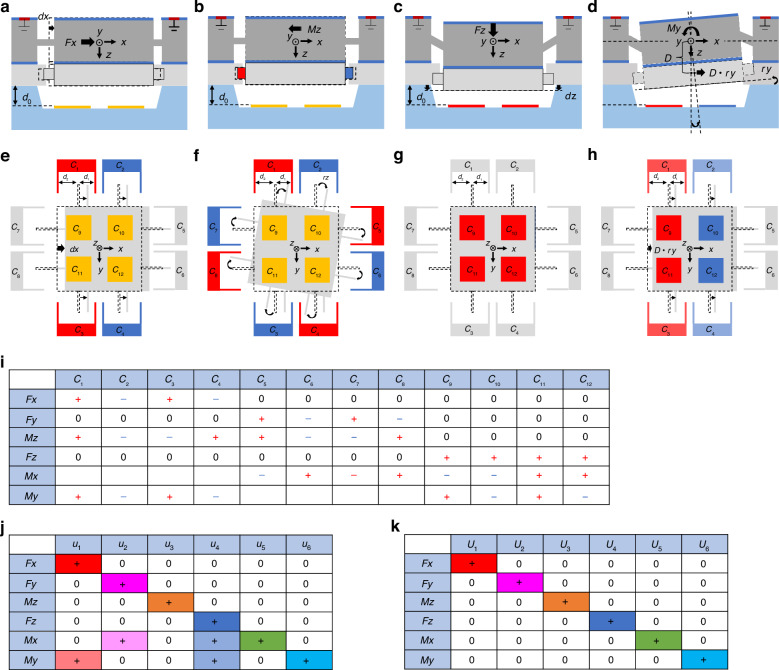
Decoupling principle of six-axis force/torque. **a, e** Deformation schematic of chip under *Fx*. **b, f** Deformation schematic of chip under *Mz*. **c, g** Deformation schematic of chip under *Fz*. **d, h** Deformation schematic of chip under *My*. **i** Changes in 12 capacitances under six-axis force/torque. **j, k** Changes of different decoupling formula outputs under six-axis force/torque

The crosstalk between the in-plane and out-of-plane loads is suppressed significantly by a layered design of two types of capacitors. Force *Fx* causes in-plane translational displacement *dx* of proof mass M2 and only results in a significant change in comb capacitances *C*_1_–*C*_4_ because of the variation in comb spaces *d*_1_ and *d*_2_ (Fig. 2a, e). Torque *Mz* causes in-plane rotational angle *rz* of proof mass M2 and only results in a significant change in parallel capacitances *C*_9_–*C*_12_ because of the variations in *d*_1_ and *d*_2_ (Fig. 2b, f). Force *Fz* causes out-of-plane translational displacement *dz* of proof mass M2 and only results in a significant change in parallel capacitances *C*_9_–*C*_12_ because of the variation of parallel capacitor space *d*_0_ (Fig. 2c, g); here, the change in comb capacitances *C*_1_–*C*_8_ can be suppressed by the height-difference design. Torque *My* causes out-of-plane rotational angle *ry* and significantly changes parallel capacitances *C*_9_–*C*_12_ because of the variation of space *d*_0_ (Fig. 2d, h); here, another translational displacement *D*·*ry* along the *x*-direction occurs because of eccentricity *D* and causes crosstalk for the detection of *Fx*. Figure 2i shows the detailed changes in capacitances *C*_1_–*C*_12_ under a six-axis force/torque. They reflect a high degree of symmetry. The initial decoupling of the in-plane and out-of-plane loads can be realized by detecting *Fx*, *Fy*, and *Mz* through comb capacitors *C*_1_–*C*_8_ and *Fz*, *Mx*, and *My* through parallel capacitors *C*_9_–*C*_12_.

The crosstalk within the in-plane or out-of-plane loads can be suppressed by the summation or difference decoupling operation in Eq. (1) because of the highly symmetrical arrangement of the 12 capacitors. Figure 2j shows the changes of decoupling outputs *u*_1_–*u*_6_ under six-axis force/torque. Not only *u*_6_ but also *u*_1_ and *u*_4_ change with torque *My*, causing crosstalk of torque *My* in the detection of forces *Fx* and *Fz*. Similarly, torque *Mx* causes crosstalk in the detection of forces *Fy* and *Fz*.

1 $$\left\{\begin{array}{l}{u}_{1}=+{C}_{1}-{C}_{2}+{C}_{3}-{C}_{4}\\ {u}_{2}=+{C}_{5}-{C}_{6}+{C}_{7}-{C}_{8}\\ {u}_{3}=+{C}_{1}-{C}_{2}-{C}_{3}+{C}_{4}+{C}_{5}-{C}_{6}-{C}_{7}+{C}_{8}\\ {u}_{4}=+{C}_{9}+{C}_{10}+{C}_{11}+{C}_{12}-4{C}_{R}\\ {u}_{5}=-{C}_{9}-{C}_{10}+{C}_{11}+{C}_{12}\\ {u}_{6}=+{C}_{9}-{C}_{10}+{C}_{11}-{C}_{12}\end{array}\right.$$

where *C*_*R*_ is the initial parallel-capacitor value.

The crosstalk of torque *Mx* or *My* in the detection of forces *Fx* or *Fy* is caused by the eccentricity between top and bottom silicon layers. The theoretical relationship between the decoupling outputs *u*_*1*_–*u*_*6*_ and the six-axis force/torque *Fx*–*My* can be approximated by the first-order linear term, as shown in Eq. (2).

2 $$\left[\begin{array}{c}{u}_{1}\\ {u}_{2}\\ {u}_{3}\\ {u}_{4}\\ {u}_{5}\\ {u}_{6}\end{array}\right]\approx \left[\begin{array}{cccccc}{s}_{11}{{k}_{11}}^{-1} & 0 & 0 & 0 & 0 & {s}_{11}D{{k}_{66}}^{-1}\\ 0 & {s}_{22}{{k}_{22}}^{-1} & 0 & 0 & -{s}_{22}D{{k}_{55}}^{-1} & 0\\ 0 & 0 & {s}_{33}{{k}_{33}}^{-1} & 0 & 0 & 0\\ 0 & 0 & 0 & {s}_{44}{{k}_{44}}^{-1} & 0 & 0\\ 0 & 0 & 0 & 0 & {s}_{55}{{k}_{55}}^{-1} & 0\\ 0 & 0 & 0 & 0 & 0 & {s}_{66}{{k}_{66}}^{-1}\end{array}\right]\left[\begin{array}{c}Fx\\ Fy\\ Mz\\ Fz\\ Mx\\ My\end{array}\right]$$

where *k*_*ii*_ (*i* = 1–6) is the six-degree-of-freedom stiffness of the mass-spring system consisting of S-shaped beams and proof mass M2. Here, *s*_*ii*_ (*i* = 1–6) are the first-order linear sensitivities of decoupling outputs *u*_1_–*u*_6_ to six-axis displacements/angles *dx*–*dy* of proof mass M2, respectively; *D* is the eccentricity between proof masses M1 and M2.

The complete analytical formulas of *u*_1_–*u*_6_ with *Fx*–*My* are shown in Eq. (3), which can be written as Taylor expansions, as shown in Eq. (4).

3 $$\left\{\begin{array}{l}{u}_{1}(Fx)=2\times \left(\frac{N\varepsilon {t}_{0}{l}_{0}}{{d}_{1}\,-\,{k}_{11}^{-1}Fx}+\frac{N\varepsilon {t}_{0}{l}_{0}}{{d}_{2}\,+{\,k}_{11}^{-1}Fx}\right)-2\times \left(\frac{N\varepsilon {t}_{0}{l}_{0}}{{d}_{1}\,+\,{k}_{11}^{-1}Fx}+\frac{N\varepsilon {t}_{0}{l}_{0}}{{d}_{2}\,-\,{k}_{11}^{-1}Fx}\right)\\ {u}_{2}(Fy)=2\times \left(\frac{N\varepsilon {t}_{0}{l}_{0}}{{d}_{1}\,-\,{k}_{22}^{-1}Fy}+\frac{N\varepsilon {t}_{0}{l}_{0}}{{d}_{2}\,+\,{k}_{22}^{-1}Fy}\right)-2\times \left(\frac{N\varepsilon {t}_{0}{l}_{0}}{{d}_{1}\,+\,{k}_{22}^{-1}Fx}+\frac{N\varepsilon {t}_{0}{l}_{0}}{{d}_{2}\,-\,{k}_{22}^{-1}Fy}\right)\\ {u}_{3}(Mz)\approx 4\times \left(\frac{N\varepsilon {t}_{0}{l}_{0}}{{d}_{1}\,-\,{a}_{1}\,\cdot \,{k}_{33}^{-1}Mz}+\frac{N\varepsilon {t}_{0}{l}_{0}}{{d}_{2}\,+\,{a}_{1}\,\cdot \,{k}_{33}^{-1}Mz}\right)-4\times \left(\frac{N\varepsilon {t}_{0}{l}_{0}}{{d}_{1}\,+\,a\,\cdot \,{k}_{33}^{-1}Mz}+\frac{N\varepsilon {t}_{0}{l}_{0}}{{d}_{2}\,-\,{a}_{1}\,\cdot \,{k}_{33}^{-1}Mz}\right)\\ {u}_{4}(Fz)=4\times \frac{\varepsilon {b}^{2}}{{d}_{0}\,-\,{k}_{44}^{-1}Fz}\\ {u}_{5}(Mx)\approx 2\times \frac{\varepsilon {b}^{2}}{{d}_{0}\,-\,{a}_{0}{k}_{55}^{-1}Mx}-2\times \frac{\varepsilon {b}^{2}}{{d}_{0}\,+\,{a}_{0}{k}_{55}^{-1}Mx}\\ {u}_{6}(My)\approx 2\times \frac{\varepsilon {b}^{2}}{{d}_{0}\,-\,{a}_{0}{k}_{66}^{-1}My}-2\times \frac{\varepsilon {b}^{2}}{{d}_{0}\,+\,{a}_{0}{k}_{66}^{-1}My}\end{array}\right.$$

4 $$\left\{\begin{array}{c}{u}_{1}(Fx)=\frac{4N\varepsilon {t}_{0}{l}_{0}}{{d}_{1}}\left[\left(1-\frac{{d}_{1}^{2}}{{d}_{2}^{2}}\right)\frac{1}{{d}_{1}}\frac{Fx}{{k}_{11}}+\left(1-\frac{{d}_{1}^{4}}{{d}_{2}^{4}}\right)\frac{1}{{d}_{1}^{3}}\frac{F{x}^{3}}{{k}_{11}^{3}}+\cdots \right]\\ {u}_{2}(Fy)=\frac{4N\varepsilon {t}_{0}{l}_{0}}{{d}_{1}}\left[\left(1-\frac{{d}_{1}^{2}}{{d}_{2}^{2}}\right)\frac{1}{{d}_{1}}\frac{Fy}{{k}_{22}}+\left(1-\frac{{d}_{1}^{4}}{{d}_{2}^{4}}\right)\frac{1}{{d}_{1}^{3}}\frac{F{y}^{3}}{{k}_{22}^{3}}+\cdots \right]\\ {u}_{3}(Mz)\approx \frac{8N\varepsilon {t}_{0}{l}_{0}}{{d}_{1}}\left[\left(1-\frac{{d}_{1}^{2}}{{d}_{2}^{2}}\right)\frac{{a}_{1}}{{d}_{1}}\frac{Mz}{{k}_{33}}+\left(1-\frac{{d}_{1}^{4}}{{d}_{2}^{4}}\right)\frac{{a}_{1}^{3}}{{d}_{1}^{3}}\frac{M{z}^{3}}{{k}_{33}^{3}}+\cdots \right]\\ {u}_{4}(Fz)=\frac{4\varepsilon {b}^{2}}{{d}_{0}}\left(\frac{1}{{d}_{0}}\frac{Fz}{{k}_{44}}+\frac{1}{{d}_{0}^{2}}\frac{F{z}^{2}}{{k}_{44}^{2}}+\frac{1}{{d}_{0}^{3}}\frac{F{z}^{3}}{{k}_{44}^{3}}+\cdots \right)\\ {u}_{5}(Mx)\approx \frac{4\varepsilon {b}^{2}}{{d}_{0}}\left(\frac{{a}_{0}}{{d}_{0}}\frac{Mx}{{k}_{55}}+\frac{{a}_{0}^{3}}{{d}_{0}^{3}}{\frac{Mx}{{k}_{55}^{3}}}^{3}+\cdots \right)\\ {u}_{6}(My)\approx \frac{4\varepsilon {b}^{2}}{{d}_{0}}\left(\frac{{a}_{0}}{{d}_{0}}\frac{My}{{k}_{66}}+\frac{{a}_{0}^{3}}{{d}_{0}^{3}}{\frac{My}{{k}_{66}^{3}}}^{3}+\cdots \right)\end{array}\right.$$

where ε is the permittivity of air (~8.854 × 10^−12^ F/m), the actual capacitance is replaced by the value of virtual parallel capacitors at the center of the electrode plate when the capacitors are deflected under torque, and *a*_1_ is the parameter describing the planar location of the combs from the chip origin, equal to *a* + *l*_c_ + *l*_0_/2.

The crosstalks caused by torques *Mx* and *My* in the detection of force *Fz* are caused by the higher-order nonlinear terms of *u*_4_ with *Mx* and *My*, which are ignored in Eq. (2). The complete analytical formulas are expressed by Taylor expansion, as shown in Eq. (4).

5 $$\left\{\begin{array}{l}{u}_{4}(Mx)\approx \frac{2\varepsilon {b}^{2}}{{d}_{0}-{k}_{55}^{-1}Mx}+\frac{2\varepsilon {b}^{2}}{{d}_{0}\,+\,{k}_{55}^{-1}My}=\frac{4\varepsilon {b}^{2}}{{d}_{0}}\left(\frac{{{a}_{0}}^{2}}{{{d}_{0}}^{2}}\cdot \frac{M{x}^{2}}{{{k}_{55}}^{2}}+\frac{{{a}_{0}}^{4}}{{{d}_{0}}^{4}}\cdot \frac{M{x}^{4}}{{{k}_{55}}^{4}}+\frac{{{a}_{0}}^{6}}{{{d}_{0}}^{6}}\cdot \frac{M{x}^{6}}{{{k}_{55}}^{6}}+\cdots \right)\\ {u}_{4}(My)\approx \frac{2\varepsilon {b}^{2}}{{d}_{0}-{k}_{44}^{-1}My}+\frac{2\varepsilon {b}^{2}}{{d}_{0}\,+\,{k}_{44}^{-1}My}=\frac{4\varepsilon {b}^{2}}{{d}_{0}}\left(\frac{{{a}_{0}}^{2}}{{{d}_{0}}^{2}}\cdot \frac{M{y}^{2}}{{{k}_{66}}^{2}}+\frac{{{a}_{0}}^{4}}{{{d}_{0}}^{4}}\cdot \frac{M{y}^{4}}{{{k}_{66}}^{4}}+\frac{{{a}_{0}}^{6}}{{{d}_{0}}^{6}}\cdot \frac{M{y}^{6}}{{{k}_{66}}^{6}}+\cdots \right)\end{array}\right.$$

A functional relationship exists between second-order terms *u*_4_(*Mx*) and *u*_4_(*My*) in Eq. (5) and first-order terms of *u*_5_(*Mx*) and *u*_6_(*My*) in Eq. (4). The crosstalk *u*_4_(*Mx*) and *u*_4_(*My*) in Eq. (5) can be approximately expressed by *u*_5_(*Mx*) and *u*_6_(*My*) in Eq. (4), respectively, as shown in Eq. (6).

6 $$\left\{\begin{array}{l}{u}_{4}(Mx)\approx \frac{{{u}_{5}}^{2}(Mx)}{4{C}_{R}}\\ {u}_{4}(My)\approx \frac{{{u}_{6}}^{2}(My)}{4{C}_{R}}\end{array}\right.$$

By analyzing the theoretical model of the crosstalk terms, the crosstalk caused by the eccentricity *D* in Eq. (2) and that caused by nonlinearity in Eq. (6) can be compensated for by the decoupling operation, as shown in Eq. (7). Figure 2k shows the changes in the new decoupling outputs *U*_1_–*U*_6_ under six-axis force/torque, which have better decoupling ability in theory than *u*_1_–*u*_6_.

7 $$\begin{array}{l}\left[\begin{array}{c}{U}_{1}\\ {U}_{2}\\ {U}_{3}\\ {U}_{4}\\ {U}_{5}\\ {U}_{6}\end{array}\right]=\left[\begin{array}{c}{u}_{1}-{k}_{1}D{{s}_{66}}^{-1}{s}_{11}{u}_{6}\\ {u}_{2}+{k}_{2}D{{s}_{55}}^{-1}{s}_{22}{u}_{5}\\ {u}_{3}\\ {u}_{4}-({k}_{3}{u}_{5}^{2}+{k}_{4}{u}_{6}^{2})/4{C}_{R}\\ {u}_{5}\\ {u}_{6}\end{array}\right]\\\approx \left[\begin{array}{cccccc}{s}_{11}{{k}_{11}}^{-1} & 0 & 0 & 0 & 0 & 0\\ 0 & {s}_{22}{{k}_{22}}^{-1} & 0 & 0 & 0 & 0\\ 0 & 0 & {s}_{33}{{k}_{33}}^{-1} & 0 & 0 & 0\\ 0 & 0 & 0 & {s}_{44}{{k}_{44}}^{-1} & 0 & 0\\ 0 & 0 & 0 & 0 & {s}_{55}{{k}_{55}}^{-1} & 0\\ 0 & 0 & 0 & 0 & 0 & {s}_{66}{{k}_{66}}^{-1}\end{array}\right]\left[\begin{array}{c}Fx\\ Fy\\ Mz\\ Fz\\ Mx\\ My\end{array}\right]\end{array}$$

where *k*_1_–*k*_4_ are correction coefficients considering the errors caused by the theoretical approximation and actual factors.

In general, decoupled detection for six-axis force/torque can be realized in principle, through the decoupling structure design and decoupling operation of Eqs. (1) and (7).

### Optimization of capacitors

The size parameters of the capacitors are optimized to improve the displacement/angle sensitivity. The first-order linear displacement/angle sensitivity *s*_ii_ (*i* = 1–6) in Eqs. (2) and (7) can be obtained using the Taylor expansions in Eq. (4), as in Eq. (8).

8 $$\left\{\begin{array}{l}{s}_{11}={s}_{22}=4N\varepsilon {t}_{0}{l}_{0}\left(\frac{1}{{{d}_{1}}^{2}}-\frac{1}{{{d}_{2}}^{2}}\right)\\ {s}_{33}\approx 8N\varepsilon {t}_{0}{l}_{0}\left(\frac{1}{{{d}_{1}}^{2}}-\frac{1}{{{d}_{2}}^{2}}\right)\cdot {a}_{1}\\ {s}_{44}=\frac{4\varepsilon {b}^{2}}{{{d}_{0}}^{2}}\\ {s}_{55}={s}_{66}\approx \frac{4\varepsilon {b}^{2}}{{{d}_{0}}^{2}}\cdot {a}_{0}\end{array}\right.$$

where *N* is not an independent parameter and is correlated with *a*, *d*_1_, *d*_2_, and *w*, as in Eq. (9).

9 $$N\approx \frac{a}{{d}_{1}+{d}_{2}+2w}$$

**Fig. 3:**
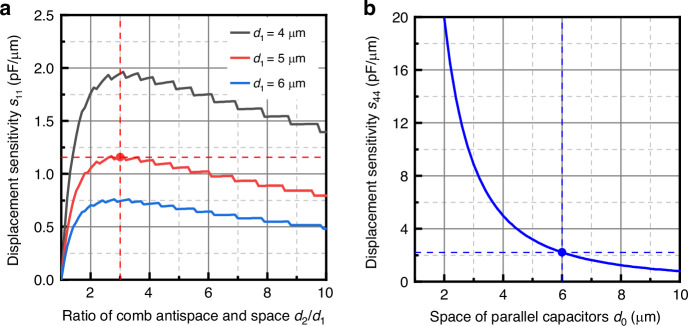
Optimization of displacement sensitivity. **a** Variation of *s*_11_ with space *d*_1_ and antispace *d*_2_ of comb capacitors. **b** Variation of *s*_44_ with space *d*_0_ of parallel capacitors

Figure 3a shows the variation of sensitivity *s*_11_ with comb space *d*_1_ and antispace *d*_2_. Here, parameters *a*, *t*_0_, and *l*_0_ are taken as large as possible, and *w* is taken as small as possible, considering the limitations of the overall size, layout, and fabrication of the chip. The final values are listed in Table 1. Sensitivity *s*_11_ increases with an increase in space *d*_1_, and an optimal extreme point exists with an increase in antispace *d*_2_. Finally, considering the DRIE process, comb space *d*_1_ and antispace *d*_2_ are taken as 5 and 15 μm, respectively.

Figure 3b shows the variation in sensitivity *s*_44_ with parallel capacitor space *d*_0_. Here, *b* and *a*_*0*_ are taken as large as possible considering the limitations of the chip overall size and layout. Sensitivity *s*_44_ decreases with increasing space *d*_0_, and *d*_0_ is taken as 6 μm considering the anodic bonding process.

### Supporting beam structure optimization

The sensing-chip measuring range and sensitivity are determined by the supporting-beams stiffness and strength after the capacitor sizes are determined. The S-shaped beams are designed and optimized to improve the chip range because silicon-based MEMS chips tend to have poor load-bearing capacity because they are small and fragile.

Two types of S-shaped beam are designed and symmetrically arranged at the corner and middle positions around proof mass M2 to increase the effective length in a limited layout space and improve the stress–dispersion characteristics. The corner and middle S-shaped beams have three independent size parameters (Fig. 1b and Table 1).

The S-shaped beam parameters are optimized by static solid mechanics simulation using COMSOL simulation software to obtain the maximum range within the silicon stress limit.

**Fig. 4:**
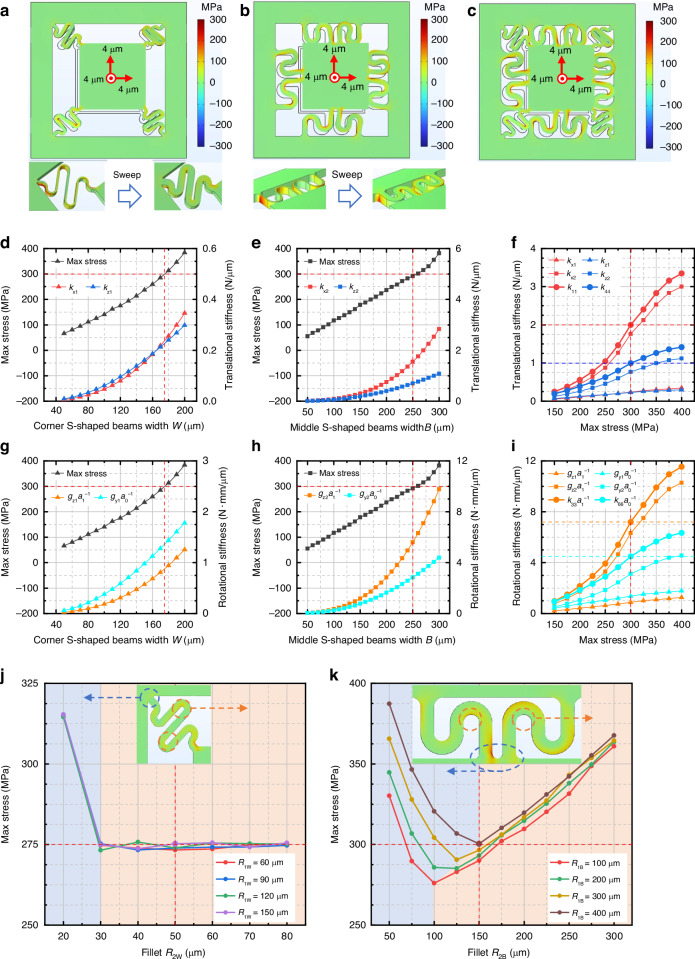
Optimization of S-shaped beams. **a** Finite-element model of corner S-shaped beams. **b** Finite-element model of middle S-shaped beams. **c** Finite-element model of S-shaped beams in parallel. **d, g** Stiffness and full-range-state maximum stresses of corner S-shaped beams under different widths *W.* **e, h** Stiffness and full-range-state maximum stress of middle S-shaped beams under different widths *B.* **f, i** Stiffness that can be achieved by S-shaped beams under different full-range stress levels. **j, k** Analysis of the influence of fillets on the full-range-state maximum stress

First, the full-range-state maximum stress for the two types of S-shaped beam under different widths are calculated by parametric sweep studies (Fig. 4a and b, respectively). The full-range states are realized by applying the allowable maximum displacements (4 μm, 4 μm, and 4 μm) along the *x*-, *y*-, and *z*-directions to proof mass M2, respectively. Figure 4d, g shows the variation of maximum stress with the width *W* of the corner S-shaped beams, and Fig. 4e, h shows the variation in the maximum stress with the width *B* of the middle S-shaped beams.

Second, the six-degree-of-freedom stiffnesses of the two types of S-shaped beam under different widths are calculated in another parametric sweep study. Figure 4d, g shows the variations of the corner S-shaped beams translational and rotational stiffnesses with width *W*, respectively. Figure 4e, h shows the variations of the middle S-shaped beams translational and rotational stiffnesses with *B*, respectively. According to Fig. 4d, e, g, and h, *W* and *B* are taken as 175 and 250 μm, respectively, to obtain the maximum stiffness while satisfying the limit stress of 300 MPa under the full-range state.

Third, in another parametric sweep study, the full-range-state maximum stresses of two types of S-shaped beam under different fillets are calculated after *W* and *B* are determined. There is a transfer phenomenon in the stress concentration region with the variation of the fillets. The stress concentration region of the corner S-shaped beams is transferred from fillets *R*_1W_ to intermediate fillets with the increase of fillets *R*_2W_ (Fig. 4j). Fillets *R*_1W_ and *R*_2W_ are taken as 150 and 50 μm, respectively, so that the potential stress concentration regions have the most average and minimum stress. The stress concentration region of the middle S-shaped beams is transferred from fillets *R*_2B_ to the intermediate fillets with the increase of fillets *R*_2B_ (Fig. 4k). Fillets *R*_1B_ and *R*_2B_ are taken as 400 and 150 μm, respectively, so that the potential stress concentration regions have the most average stress near 300 MPa and the S-shaped beams can achieve the maximum stiffness.

Figure 4f, i shows the stiffnesses of different S-shaped beams under different full-range stress values. Under an ultimate stress of 300 MPa for parallel S-shaped beams, the in-plane translational stiffness *k*_11_ or *k*_22_ is approximately 2.00 N/μm, the out-of-plane translational stiffness *k*_44_ is approximately 0.99 N/μm, the in-plane rotational stiffness *k*_33_·*a*_1_^−1^ is approximately 7.20 N·mm/μm, and the out-of-plane rotational stiffness *k*_66_·*a*_0_^−1^ is approximately 4.48 N·mm/μm.

Table 1 lists the optimized size values, and Table 2 shows the theoretical chip performance. Here, the *Fx*/*Fy*, *Mz*, *Fz*, and *Mx*/*My* ranges can reach 2.5 N, 12.5 N·mm, 2.5 N·mm, and 5 N·mm, respectively. The full-range displacements of the capacitor center positions are 1.25 μm, 1.7 μm, 2.5 μm, and 1.1 μm, respectively, which are smaller than the capacitor initial space values, to ensure good linearity. An overload capacity of 200%FS can then be achieved.

**Table 2:** Theoretical performances of optimized chip

Index	*Fx* or *Fy*	*Mz*	*Fz*	*Mx* or *My*
Displacement sensitivity	1.16 pF/μm	2.32 pF/μm	2.21 pF/μm	2.21 pF/μm
Stiffness	2.00 N/μm	7.20 N·mm/μm	0.99 N/μm	4.48 N·mm/μm
Full-range force/torque	2.5 N	12.5 N·mm	2.5 N	5.0 N·mm
Full-range dis/angle	1.25 μm	1.7 μm	2.5 μm	1.1 μm
Over force/torque	200%FS	200%FS	200%FS	200%FS
Theoretical sensitivity	0.58 pF/N	0.32 pF/(N·mm)	2.23 pF/N	0.49 pF/(N·mm)
Test sensitivity	0.52 pF/N	0.27 pF/(N·mm)	2.87 pF/N	0.44 pF/(N·mm)

## Results and discussion

### Finite-element model results

**Fig. 5:**
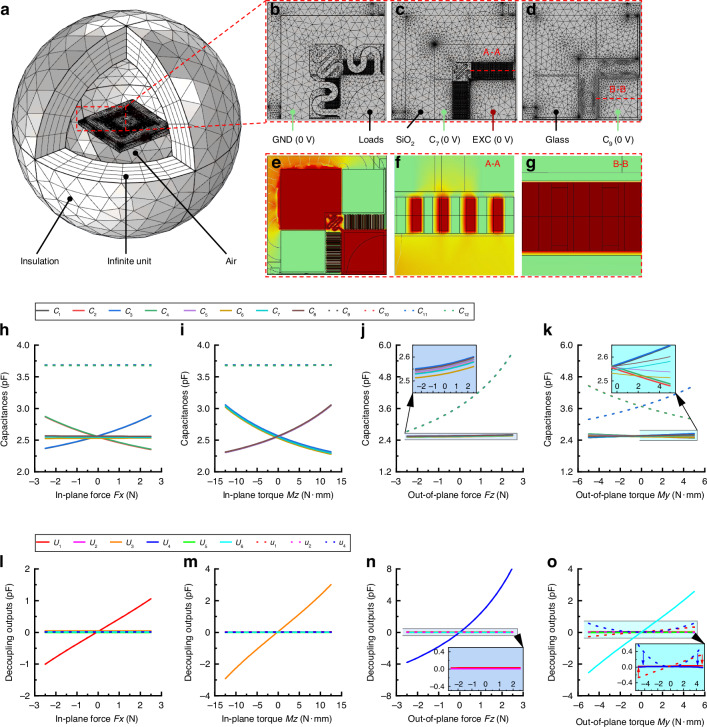
Finite-element simulation. **a** Schematic of overall model. **b** Mesh and boundary conditions on different chip structures. **c** Potential distribution under specific load. **d** The 12 capacitances outputs under different loads. **e–g** Potential distribution at different chip locations. **h–o** Decoupling outputs under different loads

A solid mechanics and electrostatically coupled multiphysics field finite-element model was developed using COMSOL to verify the chip design, principle, and theories discussed in Section 2. Figure 5a shows a schematic of the overall model. Figure 5b shows the mesh and boundary conditions applied to the chip structures. Ground terminal GND is applied to the top silicon, and a rigid domain to which six-axis force/torque is applied directly is set at the center of proof mass M1. Voltage terminal EXC (1 V) is applied to proof mass M2 as an excitation signal, and the other 12 voltage terminals C_1_–C_12_ (0 V) are applied to the fixed plates of combs and parallel capacitors as the output signal.

The in-plane force *Fx*, in-plane torque *Mz*, out-of-plane force *Fz*, and out-of-plane torque *My* were applied to the rigid domain. Parametric sweep studies were performed in the corresponding range, and the equivalent capacitances under different loads were calculated.

Figure 5e–g shows the simulation results of the potential distribution under a specific load. Figure 5e shows the overall layout of the potential distribution in the horizontal cross section of the bottom silicon. A potential gradient between moving and fixed plates forms for the comb and parallel capacitor (Fig. 5f and g, respectively).

Figure 5h–k shows the variations of 12 capacitances with different forces/torques. They have a high degree of symmetry, consistent with the principle analysis in Section 2.1. The simulation results also verify that comb capacitances *C*_1_, *C*_3_ increase and *C*_2_, *C*_4_ decrease with increasing *My*, as shown in the zoom-in view in Fig. 5k, because *My* causes an additional displacement of proof mass M1 along the *x*-direction because of eccentricity *D*.

The 12 capacitances in Fig. 5h–k are introduced into Eqs. (1) and (7) for the decoupling operation. Figure 5l–o shows the variations in the decoupling output with different forces/torques. A good correspondence and low crosstalk were obtained. Torque *My* results in the obvious crosstalk outputs of *u*_1_ and *u*_4_ (dotted blue and red lines in Fig. 5k), and the crosstalk outputs of *U*_1_ and *U*_4_ can be suppressed to nearly zero (blue and red lines in Fig. 5k). The finite-element model verified the correctness of the coupling and decoupling mechanisms caused by the eccentricity and the nonlinear effect discussed in Section 2.1.

### Fabrication and packaging

**Fig. 6:**
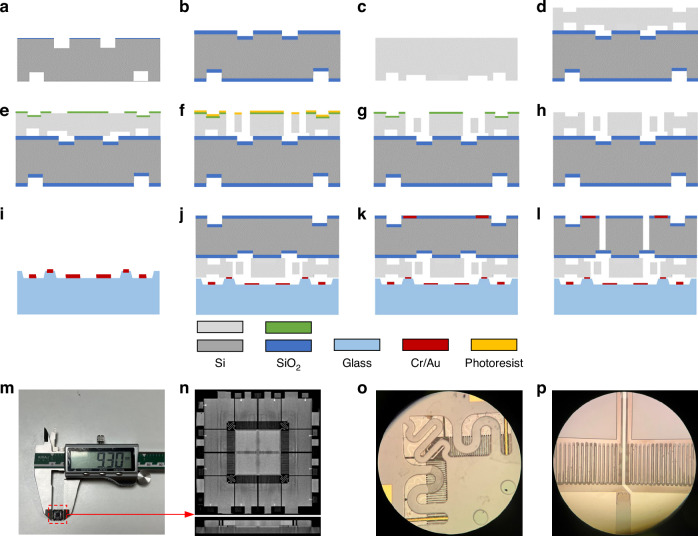
Chip fabrication processes. **a** RIE for mark and cavity. **b** Thermal oxidization. **c** RIE for mark and height difference. **d** Si–Si bonding, thinning, polishing, and RIE for mark. **e** CVD SiO_2_ mask and patterning. **f** Photolithography and DRIE for combs. **g** RIE for height difference. **h** SiO_2_ mask removed by vapor HF. **i** Glass etching and metal patterning. **j** Anodic bonding. **k** SiO_2_ window opening and metal patterning. **l** DRIE for S-shaped beams. **m** Single chip after scribing. **n** CT scan of overall chip. **o** Micrograph of S-shaped beams. **p** Micrograph of capacitors

The chip was fabricated using two 4-inch single-crystal low-resistance silicon wafers and a BF_33_ glass wafer. The fabrication process is shown in Fig. 6a–l. The process can be divided into three parts. First, a cavity silicon-on-insulator (C-SOI) wafer with internal cavities is fabricated by reactive ion etching (RIE), thermal oxygenation, Si–Si bonding, thinning, and polishing (Fig. 6a–d). Then, comb capacitors with height differences are fabricated by double-mask self-alignment, RIE, and DRIE (Fig. 6e–h). Finally, parallel capacitors and S-shaped beams are fabricated by wet etching of glass, corrosion of metal, anodic bonding, and DRIE (Fig. 6i–l).

- Alignment markers and a 10-μm cavity are etched by RIE on the 400-μm top silicon wafer with a resistivity of 0.002–0.004 Ω·cm.
- The 2-μm SiO_2_ insulating layer is fabricated on the surface of the top silicon wafer by thermal oxidation.
- The 6-μm-height-difference comb capacitors are etched by RIE on the 300-μm bottom silicon wafer with a resistivity of 0.002–0.004 Ω·cm.
- The top silicon and bottom silicon are bonded together by Si–Si bonding, and then the bottom silicon is thinned to 80 μm and polished by chemical–mechanical polishing.
- A 500-nm SiO_2_ layer is deposited on the bottom silicon by chemical vapor deposition (CVD) and patterned as the first mask.
- Photolithography on the first SiO_2_ mask and etching of the exposed SiO_2_ with a photoresist mask are performed to form a self-aligned SiO_2_-photoresist composite mask. Then, the comb capacitors are etched by DRIE.
- The second photoresist mask is removed; then, the 6-μm-height-difference comb capacitors are etched by RIE.
- The first SiO_2_ mask is removed by vapor hydrofluoric-acid (HF) etching.
- A 6-μm cavity is wet etched on the surface of a 500-μm glass wafer using a buffered oxide etchant solution; then, the patterned electrodes, leads, and pads are fabricated on the surface by metal deposition and corrosion process.
- The C-SOI and glass wafers are bonded together by anodic bonding.
- An opening window on SiO_2_ is fabricated on the surface of the top silicon wafer. Grounded pads are fabricated on the open window region by metal deposition and corrosion processes.
- Photolithography on the surface of the top silicon wafer and etching of the exposed SiO_2_ are performed using a photoresist mask; then, the S-shaped beams are etched by DRIE.

Figure 6m shows the single chip after scribing, with the size of 9.3 × 9.3 × 0.98 mm (length × width × height). Micrographs of the overall chip, S-shaped beams, and capacitors are shown in Fig. 6n–p, respectively.

### Experimental test results

**Fig. 7:**
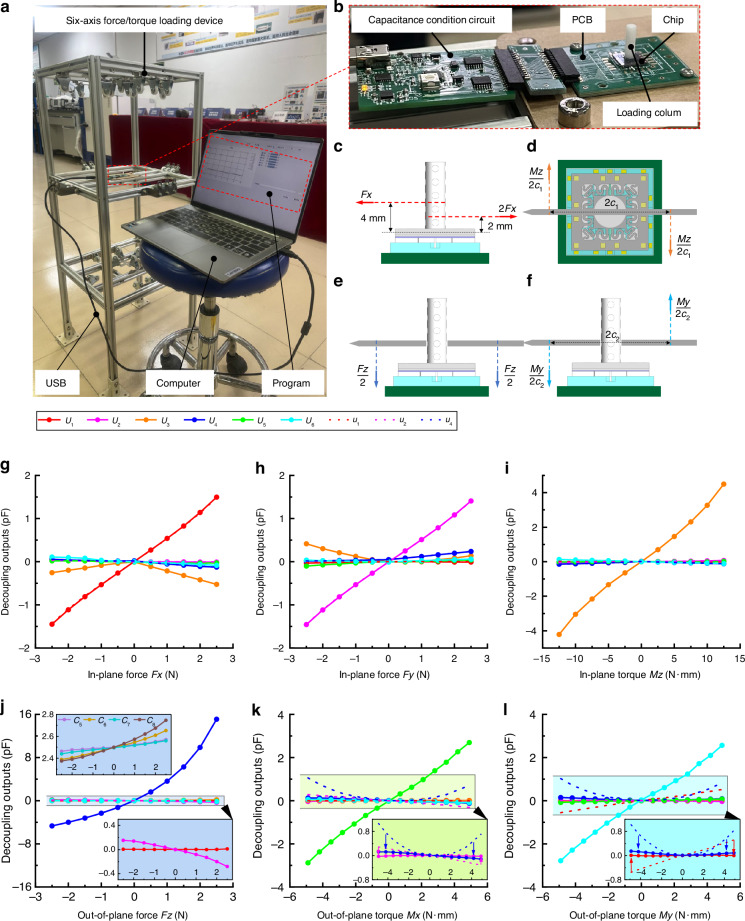
Loading test of chip. **a, b** Composition of the loading and testing system. **c–f** Schematic diagrams of loading principles for different forces/torques. **g–l** Test curves of decoupled outputs with six-axis force/torque

Figure 7a, b shows the loading and testing system. Six-axis force/torque loading equipment that uses standard weights as the load source was built. Six-axis force/torque loading can be achieved by switching the direction through tension lines and fixed pulleys. The chip glued to the printed circuit board is fixed at the center of the loading device and connected to the multichannel capacitance condition circuit, USB, and computer to transmit electrical signals.

A loading column with positioning holes is bonded on the chip surface (Fig. 7b). The six-axis force/torque is applied to the chip through the column or the rigid pin passing through the column. Figure 7c–f shows the loading principles of *Fx*, *Mz*, *Fz*, and *My*, respectively. Torque *Mz* is loaded with a force arm length *c*_1_ of 50 mm, and torques *Mx* and *My* are loaded with a force arm length *c*_2_ of 70 mm. Forces *Fx* and *Fy* are loaded in steps of 0.5 N to 2.5 N, torque *Mz* is loaded in steps of 2.5 N·mm to 12.5 N·mm, force *Fz* is loaded in steps of 0.5 N to 2.5 N, and torque *My* is loaded in steps of 0.7 N·mm to 4.9 N·mm.

Figure 7g–l shows the variations of decoupling outputs with *Fx*, *Fy*, *Mz*. *Fz*, *Mx*, and *My*, respectively. The chip has excellent decoupling detection capacity for six-axis force/torque. The six-channel decoupled outputs *U*_1_–*U*_6_ show a significant response only to *Fx*–*My*, respectively. Here, the simulation full range outputs in Fig. 5l–o are larger than the test results in Fig. 7g–j because the widths of S-shaped beams are smaller than the design values owing to the linewidth loss during the DRIE in Fig. 6l. The smaller beam widths will result in smaller stiffness and larger capacitance change under the same force/torque. In addition, each force/torque causes a small crosstalk to the outputs *U*_1_–*U*_6_ used for the detection of other forces/torques.

The test curves also show that the out-of-plane torques *Mx* and *My* cause crosstalk for *u*_2_, *u*_4_ and *u*_1_, *u*_4_ (Fig. 7k and l, respectively). The crosstalk can be significantly suppressed when new decoupling outputs *U*_1_, *U*_2_, and *U*_4_ are used. The correction coefficients *k*_1_–*k*_4_ in Eq. (7) are 1.3, 0.3, 1.5, and 1.5, respectively.

The test sensitivities for the detection of *Fx*, *Fy*, *Mz*, *Fz*, *Mx*, and *My* are approximately 0.53 pF/N, 0.52 pF/N, 0.27 pF/(N·mm), 2.87 pF/N, 0.48 pF/(N·mm), and 0.44 pF/(N·mm), respectively, which are closer to the theoretical values in Table 2. Table 3 lists all 30 crosstalk errors calculated using the simulation and test data. Except for the crosstalk between *Fz* and *Fy* (Fig. 7j), the values are all less than 6%FS, and 18 errors are lower than 2%FS. The simulation crosstalk errors are much smaller and less than 1%FS when *k*_1_–*k*_4_ are 0.8, 0.8, 1.3, and 1.3, respectively.

**Table 3:** Comparison of crosstalk errors between simulation and test

	*U*_1_(%FS)	*U*_2_(%FS)	*U*_3_(%FS)	*U*_4_(%FS)	*U*_5_(%FS)	*U*_6_(%FS)
***Fx***	/	1.45	6.00 (0.02)	0.92 (0.01)	0.95	3.60 (0.05)
***Fy***	1.11	/	4.87	1.13	2.51	1.25
***Mz***	0.57 (0.08)	3.12	/	0.80 (0.04)	0.59	3.54 (0.01)
***Fz***	0.35 (0.10)	15.1	2.05 (0.22)	/	3.64	3.26 (0.22)
***Mx***	0.79	1.09	0.23	1.24	/	2.83
***My***	0.80 (0.89)	1.22	0.87 (0.04)	0.69 (0.20)	2.71	/

The largest crosstalk of 15.1%FS occurs in the coupling output of *U*_2_, which is caused the asymmetric change of comb capacitances *C*_5_ − *C*_8_ under out-of-plane force *Fz*, as shown in the two zoomed-in view of Fig. 7j. The asymmetric change of *C*_5_ − *C*_8_ in Fig. 7j is inconsistent with the simulation analysis in Fig. 5j. What’s more, the tested coupled output of *U*_2_ (15.1%FS) and *U*_1_ (0.35%FS) in Fig. 7j is not at the same level, which is also inconsistent with the simulation analysis in Fig. 5n and the completely symmetric design concept. The inconsistency between the test and design can be caused by the tilt angle error of the chip around the *x*-axis in practice, which will cause additional in-plane force *Fy* under out-of-plane force *Fz* and result in the significant coupled outputs of *U*_2_.

The matrix fitting decoupling method can further suppress the large crosstalk errors and nonlinearity. Here, six six-element high-order fitting functions containing 50 independent parameters were established, as shown in Eq. (10). The function terms inside parentheses are used to improve the linearity of the output, and the other terms are mainly used to suppress the crosstalk. The quadratic terms of both *U*_1_ and *U*_2_ are introduced in the equation of *U*_*Mz*_ because *Fx* and *Fy* in both positive and negative cause the in-phase coupled output of *U*_3_, as shown in Fig. 7g, h.

10 $$\left\{\begin{array}{l}{U}_{Fx}={a}_{0}+({a}_{1}{U}_{1}+{a}_{12}{U}_{1}^{2}+{a}_{13}{U}_{1}^{3})+{a}_{2}{U}_{2}+{a}_{3}{U}_{3}+{a}_{4}{U}_{4}+{a}_{5}{U}_{5}+{a}_{6}{U}_{6}\\ {U}_{Fy}={b}_{0}+{b}_{1}{U}_{1}+({b}_{2}{U}_{2}+{b}_{22}{U}_{2}^{2}+{b}_{23}{U}_{2}^{3})+{b}_{3}{U}_{3}+{b}_{4}{U}_{4}+{b}_{5}{U}_{5}+{b}_{6}{U}_{6}\\ {U}_{Mz}={c}_{0}+{c}_{1}{U}_{1}+{c}_{2}{U}_{2}+({c}_{3}{U}_{3}+{c}_{32}{U}_{3}^{2}+{c}_{33}{U}_{3}^{3})+{c}_{4}{U}_{4}+{c}_{5}{U}_{5}+{c}_{6}{U}_{6}+[{c}_{12}{U}_{1}^{2}+{c}_{22}{U}_{2}^{2}]\\ {U}_{Fz}={d}_{0}+{d}_{1}{U}_{1}+{d}_{2}{U}_{2}+{d}_{3}{U}_{3}+({d}_{4}{U}_{4}+{d}_{42}{U}_{4}^{2}+{d}_{43}{U}_{4}^{3})+{d}_{5}{U}_{5}+{d}_{6}{U}_{6}\\ {U}_{Mx}={e}_{0}+{e}_{1}{U}_{1}+{e}_{2}{U}_{2}+{e}_{3}{U}_{3}+{e}_{4}{U}_{4}+({e}_{5}{U}_{5}+{e}_{52}{U}_{5}^{2}+{e}_{53}{U}_{5}^{3})+{e}_{6}{U}_{6}\\ {U}_{My}={f}_{0}+{f}_{1}{U}_{1}+{f}_{2}{U}_{2}+{f}_{3}{U}_{3}+{f}_{4}{U}_{4}+{f}_{5}{U}_{5}+({f}_{6}{U}_{6}+{f}_{62}{U}_{6}^{2}+{f}_{63}{U}_{6}^{3})\end{array}\right.$$

Using the six-element functions in Eq. (10) to fit the test data in Fig. 7g–l, the value of 50 parameters can be obtained to minimize the crosstalk errors, as shown in Eq. (11).

11 $$\begin{array}{c}\left[\begin{array}{ccccccccccc}{a}_{0} & {a}_{1} & {a}_{12} & {a}_{13} & {a}_{2} & {a}_{3} & {a}_{4} & {a}_{5} & {a}_{6} & 0 & 0\\ {b}_{0} & {b}_{1} & {b}_{2} & {b}_{22} & {b}_{23} & {b}_{3} & {b}_{4} & {b}_{5} & {b}_{6} & 0 & 0\\ {c}_{0} & {c}_{1} & {c}_{2} & {c}_{3} & {c}_{32} & {c}_{33} & {c}_{4} & {c}_{5} & {c}_{6} & {c}_{12} & {c}_{22}\\ {d}_{0} & {d}_{1} & {d}_{2} & {d}_{3} & {d}_{4} & {d}_{5} & {d}_{51} & {d}_{52} & {d}_{6} & 0 & 0\\ {e}_{0} & {e}_{1} & {e}_{2} & {e}_{3} & {e}_{4} & {e}_{5} & {e}_{52} & {e}_{53} & {e}_{6} & 0 & 0\\ {f}_{0} & {f}_{1} & {f}_{2} & {f}_{3} & {f}_{4} & {f}_{5} & {f}_{6} & {f}_{62} & {f}_{63} & 0 & 0\end{array}\right]\\ =\left[\begin{array}{ccccccccccc}0.000 & 1.883 & -0.017 & -0.085 & -0.015 & -0.004 & -0.001 & -0.008 & -0.006 & 0 & 0\\ -0.003 & 0.028 & 1.907 & 0.021 & -0.080 & -0.025 & 0.041 & 0.005 & 0.012 & 0 & 0\\ 0.001 & 0.324 & 0.331 & 3.543 & -0.018 & -0.036 & -0.026 & 0.007 & -0.052 & 0.740 & -0.507\\ -0.006 & 0.025 & -0.030 & -0.002 & 0.377 & -0.027 & 0.001 & 0.018 & 0.005 & 0 & 0\\ 0.015 & 0.039 & -0.095 & -0.004 & 0.012 & 2.035 & 0.015 & -0.037 & -0.056 & 0 & 0\\ 0.002 & 0.153 & -0.019 & 0.059 & 0.017 & 0.057 & 2.122 & 0.029 & -0.041 & 0 & 0\end{array}\right]\end{array}$$

**Fig. 8:**
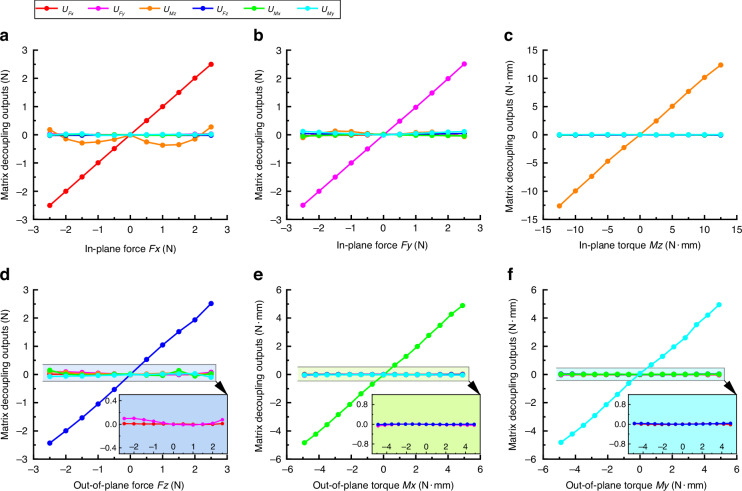
Test curves of matrix fitting decoupled outputs with six-axis force/torque. **a** In-plane force *Fx*. **b** In-plane force *Fy*. **c** In-plane torque *Mz*. **d** Out-of-plane force *Fz*. **e** Out-of-plane torque *Mx*. **f** Out-of-plane torque *My*

Further decoupling operations are carried out by the decoupling equation in Eq. (10) and fitting parameters in Eq. (11). Figure 8 shows the variation curves of matrix decoupling outputs *U*_*Fx*_ − *U*_*My*_ with six-axis force/torque. Compared with the curves in Fig. 7g–l, the matrix decoupling outputs in Fig. 8a–f have better linearity and lower crosstalk. Table 4 lists all 30 crosstalk errors of matrix fitting decoupled outputs in Fig. 8, and the maximum crosstalk errors of less than 2.59%FS can be realized.

**Table 4:** Crosstalk errors of matrix fitting decoupled outputs

	*U*_*Fx*_(%FS)	*U*_*Fy*_(%FS)	*U*_*Mz*_(%FS)	*U*_*Fz*_(%FS)	*U*_*Mx*_(%FS)	*U*_*My*_(%FS)
***Fx***	/	0.72	2.59	0.46	0.39	0.62
***Fy***	0.90	/	0.93	0.58	0.77	1.33
***Mz***	0.20	0.26	/	1.04	0.55	0.50
***Fz***	0.29	2.21	0.47	/	2.14	1.28
***Mx***	0.11	1.35	0.14	0.29	/	0.34
***My***	0.79	0.53	0.09	0.86	0.11	/

The sensing performance was also compared with those of well-developed six-axis force/torque sensing chips reported in the literature (Table 5). The sensing chip developed has a higher sensitivity while meeting the requirements of a size of less than 10 mm and a range of up to newtons and newton millimeters owing to the silicon microfabrication technology and optimization of the S-shaped beams. More-comprehensive crosstalk errors were analyzed in detail, and smaller crosstalk of less than 2.59%FS can be realized owing to the proposed symmetric decoupling structure design, decoupling theory, and matrix fitting decoupling method.

**Table 5:** Comparison of performance with other reported results

	Size (mm)	Force/Torque	Range (N or N·mm)	Sensitivity (pF/N or pF/(N·mm))	Crosstalk (%FS)
Present work	9.3 × 9.3 × 0.98	*Fx, Fy* *Fz* *Mz* *Mx, My*	2.5 2.5 12.5 5	0.53, 0.52 2.87 0.27 0.48, 0.44	0.90, 2.21 1.04 2.59 2.14, 1.33
Beyeler et al.^19,20^	10 × 9 × 0.5	*Fx, Fy* *Fz* *Mz* *Mx, My*	1 × 10^−3^ 1 × 10^−3^ 2.6 × 10^−3^ 2.6 × 10^−3^	/	/
Alveringh et al.^21^	φ9.24 × 0.5	*Fx, Fy* *Fz* *Mx, My*	2.16 2.34 5.84	0.038 0.55 0.23	/
Brookhuis et al.^22^	9 × 9 × 1	*Fx, Fy* *Fz* *Mz* *Mx, My*	10 50 25 25	0.014 1.20 0.007 0.40	/
Brookhuis et al.^23^	φ9 × 1	*Fx, Fy* *Fz* *Mz* *Mx, My*	30 6 25 25	0.028, 0.039 / 0.01 0.075, 0.15	1.1, 1.4 0.9 / 2.8, 2.5

## Conclusions

A miniaturized MEMS capacitive six-axis force/torque sensing chip with S-shaped beams, comb capacitors, and parallel capacitors was developed to realize high-accuracy decoupling detection of all-direction force/torque. The range was improved by the design and stress optimization of the S-shaped beams. Crosstalk was suppressed by the layered design and symmetric arrangement of eight combs and four parallel capacitors. The crosstalk caused by nonlinear and eccentricity effects was analyzed and then suppressed by the derived decoupling theory. The small (9.3 × 9.3 × 0.98 mm) sensing chip had a large force/torque range up to 2.5 N and 12.5 N·mm, a high force/torque sensitivity up to 0.52 pF/N and 0.27 pF/(N·mm). The chip also had an excellent decoupling capacity, with all crosstalk errors less than 2.59%FS. This chip has potential applications in robot tactile sensing and biomedical fields.
